# Transforming Breast Cancer Care: Cutting-Edge Insights and Future Directions

**DOI:** 10.3390/jcm13226697

**Published:** 2024-11-07

**Authors:** Rita De Sanctis, Paola Tiberio, Alberto Zambelli, Armando Santoro

**Affiliations:** 1 Department of Biomedical Sciences, Humanitas University, Pieve Emanuele, 20072 Milan, Italy; paola.tiberio@cancercenter.humanitas.it (P.T.); alberto.zambelli@hunimed.eu (A.Z.); armando.santoro@cancercenter.humanitas.it (A.S.); 2 Medical Oncology and Hematology Unit, IRCCS Humanitas Research Hospital, 20089 Rozzano, Italy

## 1. Introduction

Breast cancer (BC) continues to be the most prevalent malignancy affecting women globally, with 2.3 million new cases diagnosed each year, representing nearly 12% of all new cancer cases, along with 685,000 associated deaths [1,2]. Despite this alarming rise in incidence, driven partly by improvements in screening and diagnostic tools, mortality rates have steadily declined due to advancements in treatment. This paradox reflects the dual challenge of managing an increasing patient population while refining therapeutic strategies to reduce mortality and improve quality of life.

Research over the past two decades has provided unprecedented insights into the biological complexity of BC, uncovering its heterogeneity and enabling the development of personalized treatment approaches. Basic and translational studies have led to the identification of new diagnostic markers, risk factors, and therapeutic targets, aiding in the tailoring of therapies to individual patient profiles. Nevertheless, BC remains a multifaceted disease with significant challenges in clinical and research settings, including the need to better understand treatment resistance, metastatic progression, and the long-term impacts of therapy.

This editorial aims to highlight the recent advancements in BC management, particularly focusing on recent updates in early and metastatic disease settings.

## 2. Innovations in Early Breast Cancer Management

### 2.1. Immunotherapy

Recent studies have underscored the importance of immunotherapy in neoadjuvant treatment strategies. For instance, the addition of pembrolizumab to neoadjuvant chemotherapy in patients with early-stage Triple-Negative BC (TNBC) yielded a 13.6% increase in pathological complete response (pCR) rates. While translating pCR into overall survival (OS) gains remains a critical area of inquiry, it is estimated that this improvement corresponds to a 5% increase in OS at five years [3]. As we assess these results, it is essential to monitor long-term outcomes and understand which subgroups benefit most from such combinations.

Beyond the strict definition of TNBC (estrogen receptor [ER] and progesterone receptor [PgR] < 1%), non-randomized evidence has also shown promising results in early ER-low (i.e., BC with ER 1–9% expression) Human Epidermal Growth Factor Receptor 2 (HER2)-negative BC, reporting a 75% pCR rate [4]. This highlights the potential for immunotherapy to target specific BC subtypes effectively. However, further validation in larger, more diverse cohorts, as well as exploration of optimal thresholds for immunohistochemical (IHC) assays, are needed.

In addition, the Neo-Checkray trial has started to yield insights into combining chemotherapy with anti-PD-L1 agents in early ER-positive BC. Preliminary data suggest enhanced pCR rates with durvalumab in high-risk populations, yet the contributions of each therapeutic modality necessitate careful examination [5]. Balancing the benefits of intensifying treatment in non-responders with potential de-escalation strategies for early responders is paramount to fostering personalized treatment approaches.

### 2.2. CDK4/6 Inhibitors

As part of the evolving scenario of the adjuvant setting of ER-positive HER2-negative early BC (EBC), cyclin-dependent kinase 4/6 inhibitors (CDK4/6i), in conjunction with adjuvant endocrine therapy (ET), have demonstrated significant improvements in clinical outcomes for patients at high risk of recurrence, as evidenced by the results of the monarchE [6,7] and NATALEE trials [8] for abemaciclib and ribociclib, respectively. Interestingly, these trials employed different inclusion criteria to define patients at high risk of recurrence, expanding the indication of adjuvant CDK4/6i use [9,10].

In the monarchE trial, eligibility was restricted to patients with node-positive disease, categorizing them as being at a “higher risk” of recurrence compared to patients in the NATALEE trial, which encompassed a broader spectrum of tumors, including those with node-negative disease. The NATALEE trial specifically assessed the impact of adding ribociclib to standard-of-care non-steroidal aromatase inhibitors (NSAI) in patients with stage II or III hormone receptor (HR)-positive/HER2-negative EBC at risk of recurrence. The results indicated a significant improvement in invasive disease-free survival (iDFS) with the combination therapy.

At ESMO 2024, Fasching PA presented the 4-year outcomes from the NATALEE trial [11]. In the second interim efficacy analysis, with a median follow-up of 27.7 months for iDFS, 20.2% of participants had completed the planned three years of ribociclib treatment. This analysis revealed a hazard ratio of 0.748 (95% confidence interval [CI], 0.618–0.906; one-sided *p* = 0.0014). Following this, the protocol-specified final iDFS analysis, which had a median follow-up of 33.3 months, indicated that 42.8% of patients completed three years of ribociclib treatment, yielding a hazard ratio of 0.749 (95% CI, 0.628–0.892; nominal one-sided *p* = 0.0006). Notably, in a four-year landmark analysis, the combination of ribociclib and NSAI continued to exhibit both iDFS and distant DFS (DDFS) benefits over NSAI alone, with a 28.5% reduction in the risk of disease recurrence (hazard ratio, 0.715; 95% CI, 0.604–0.847). The absolute iDFS benefit increased from 2.7% at three years to 4.9% at four years, suggesting a sustained benefit beyond the completion of three years of ribociclib treatment. Furthermore, the enhanced efficacy of ribociclib in combination with NSAI was consistently observed across various subgroups and secondary endpoints. Ongoing OS follow-up continues to indicate a positive trend favoring the combination therapy (hazard ratio, 0.827; 95% CI, 0.636–1.074).

The safety profile of ribociclib remained stable with additional follow-up. Together, the results from the NATALEE trial provide compelling evidence supporting the incorporation of three years of ribociclib into the adjuvant NSAI regimen for a broad population of patients with HR-positive/HER2-negative EBC at risk of recurrence, highlighting the need for expanded indications for CDK4/6i in this setting.

### 2.3. PARP Inhibitors

The introduction of the poly ADP ribose polymerase (PARP) inhibitor olaparib in the adjuvant setting marks a paradigm shift for patients with germline BReast CAncer gene (BRCA)1 or BRCA2 pathogenic or likely pathogenic variants. In individuals with high-risk, HER2-negative EBC, the administration of adjuvant olaparib following local treatment and neoadjuvant or adjuvant chemotherapy has been associated with a significantly longer iDFS compared to placebo. Notably, the impact of olaparib on global patient-reported quality of life was limited, suggesting that its benefits are primarily focused on survival outcomes [12].

Based on the OlympiA study results [13], olaparib is indicated either as a monotherapy or in combination with endocrine therapy for the adjuvant treatment of adult patients with high-risk, HER2-negative EBC who possess germline BRCA1/2 mutations and have previously undergone neoadjuvant or adjuvant chemotherapy. Specifically, for those receiving neoadjuvant therapy, the indication applies to TNBC patients who do not achieve pCR, as well as HR-positive patients without pCR and a CPS + EG score of 3 or higher. In the adjuvant group, olaparib is recommended for TNBC patients with T2 tumors and/or N1 lymph node involvement, and for HR-positive patients with four or more positive lymph nodes.

This new algorithm enhances treatment personalization and optimizes therapeutic outcomes for patients at high risk of recurrence, underlining the importance of integrating targeted therapies into the adjuvant treatment landscape.

## 3. Advances in Metastatic Breast Cancer Therapies

Antibody–drug conjugates (ADCs) represent a revolutionary advancement in oncology, characterized by their complex and multifaceted mechanisms of action and specific treatment-related adverse events (TRAEs) [14,15,16].

The introduction of T-DXd for the treatment of HER2-low and HER2-ultralow metastatic BC (mBC) presents significant challenges regarding the determination of HER2 status in tumors from patients with HR-positive mBC. Given this evolving therapeutic landscape, all archival assessments yielding a HER2 immunohistochemistry (IHC) 0 result must undergo re-evaluation, particularly in light of the findings from the DESTINY-Breast 06 trial and the demonstrated benefits of T-DXd in HER2-low and HER2-ultralow tumors [17,18,19]. Importantly, testing HER2 status on primary or metastatic lesions has been shown to possess equivalent predictive value, emphasizing the need for accurate assessment regardless of tumor location. In cases where HER2 null or HER2 0 results are obtained, re-biopsy remains a viable option. A critical question remains regarding the efficacy of T-DXd administration in HER2 null patients. The ongoing investigation of this treatment approach, particularly through the confirmation of the DAISY trial results in the context of DESTINY-Breast 15 [20], will provide further insights into the potential applicability of T-DXd in this patient population.

To avoid the emergence of acquired resistance, current research has identified well-established in vivo resistance mechanisms primarily associated with both the target and the payload of the ADCs [21]. Importantly, the unique properties of the antibody, linker, and payload are critical factors influencing the potential for cross-resistance among different ADCs [22]. In specific clinical contexts, the sequential administration of multiple ADCs may yield effective therapeutic outcomes [23]. A systematic analysis of the distinct mechanisms underlying ADCs, combined with comprehensive evaluations of patient-derived material, is essential to accelerate the development of novel ADCs and combination therapies aimed at overcoming resistance.

In the metastatic setting, recent findings from the DESTINY Breast12 trial signify a breakthrough for HER2-positive advanced BC management. This trial reported a median progression-free survival (PFS) of 17.3 months, with favorable outcomes observed in patients with both stable and active brain metastases [24]. The efficacy in this challenging population offers new hope for patients with limited therapeutic options. However, the emergence of TRAEs, particularly interstitial lung disease (ILD) and pneumonitis, necessitates stringent monitoring.

The exploration of new therapeutic options, including other ADCs such as ESG401 and patritumab deruxtecan [25,26], further illustrates the ongoing innovation in this domain. Early results indicate promising objective response rates and PFS, suggesting that ADCs may herald a new era in targeted BC therapies. The ICARUS-BREAST01 study also highlights the potential of patritumab deruxtecan (an anti-Human Epidermal Growth Factor Receptor-3 monoclonal antibody conjugated to a topoisomerase-I inhibitor), with a median PFS of 9.4 months among HR-positive and HER2-negative patents progressing after at least two lines of therapy, underlining the importance of continued research in this area [27].

## 4. Addressing Gaps in Knowledge

Addressing the complexities of BC requires a concerted effort to bridge existing gaps in knowledge. For example, the integration of biomarker-driven strategies is essential for optimizing treatment outcomes. The determination of HER2-low and HER2-ultralow status has become increasingly relevant, particularly with the implications of trials like DESTINY-Breast06. Ensuring consistency in HER2 testing methodologies and interpretation is vital for guiding therapeutic decisions.

Moreover, ongoing research into predictive biomarkers for ADC efficacy is crucial for advancing personalized treatment. Recent studies have shown that specific mutational signatures in tumors can be associated with responses to trastuzumab deruxtecan [28], paving the way for a more tailored approach to therapy based on individual genomic profiles.

The question persists: can we leverage the heterogeneity of BC to identify optimal therapeutic combinations? Investigations into signaling pathways such as the PIK3CA/AKT1/PTEN axis in TNBC may elucidate potential subgroups that could benefit from targeted therapies, making ongoing clinical trials essential for generating actionable insights.

In this Special Issue, “Breast Cancer: Pathology, Diagnosis, and Treatment”, five papers have been published, each contributing critical knowledge to our understanding of BC’s pathology and treatment.

First, the study by Burguin A. et al. explored the effects of aminosteroid RM-581 on BC cell lines representative of all BC molecular subtypes, showing its ability to decrease cell proliferation, both alone and in combination with existing treatments [29]. This work highlights the potential for novel agents to complement standard therapies and overcome resistance.

Next, the investigation into plasma levels of MMP-3 and MMP-7 in EBC patients by Ławicki et al. identified these metalloproteinases as potential diagnostic biomarkers [30]. Their work underscores the ongoing need for non-invasive tools to improve early detection and monitoring.

The role of CDK 4/6i in managing brain metastases in BC patients was retrospectively addressed by Kubeczko M and colleagues [31], showing promising efficacy in combination with radiotherapy. This study tackles a critical gap in treatment, as brain metastases remain a significant clinical challenge with limited therapeutic options.

The impact of adverse events’ management on treatment outcomes was the focus of our pivotal study, which examined guideline-based diarrhea management in patients treated with abemaciclib [32]. This research highlights the importance of supportive care strategies in enhancing adherence to therapy and improving patient outcomes. As a further step, we also explored the use of a new postbiotic agent to prevent and reduce the severity of abemaciclib-induced diarrhea [33].

Lastly, the review by Brogowska KK and colleagues on vascular endothelial growth factor (VEGF) ligands and receptors in BC provides a comprehensive overview of their role in tumor angiogenesis and progression and suggests new avenues for therapeutic targeting in the future [34].

Collectively, these papers address critical gaps in the current understanding of BC, from novel therapeutic agents to biomarkers and supportive care strategies. Future research must continue to focus on identifying predictive biomarkers for treatment response, improving metastatic disease management, and refining strategies to mitigate long-term treatment toxicity. By bridging these knowledge gaps, we can further personalize BC care and continue to improve outcomes for patients.

## 5. Conclusions

The urgent need for collaborative efforts in BC research and treatment is more pressing than ever. The integration of immunotherapies, ADCs, and biomarker-driven approaches heralds a new frontier in BC management. However, careful consideration of safety profiles, treatment sequences, and individual patient characteristics is essential for translating these advances into tangible clinical benefits.

As we stand on the cusp of groundbreaking discoveries, it is vital to remain steadfast in our commitment to improving outcomes for all BC patients. By fostering a culture of innovation and collaboration, we can strive toward a future where BC is not merely managed, but ultimately conquered.

## Data Availability

Not applicable.
